# Methanogens at the top of the world: occurrence and potential activity of methanogens in newly deglaciated soils in high-altitude cold deserts in the Western Himalayas

**DOI:** 10.3389/fmicb.2013.00359

**Published:** 2013-12-03

**Authors:** Katrin Aschenbach, Ralf Conrad, Klára Řeháková, Jiří Doležal, Kateřina Janatková, Roey Angel

**Affiliations:** ^1^Max Planck Institute for Terrestrial MicrobiologyMarburg, Germany; ^2^Institute of Botany, Academy of Sciences of the Czech RepublicTřeboň, Czech Republic; ^3^Faculty of Science, University of South BohemiaČeské Budějovice, Czech Republic

**Keywords:** methanogens, desert ecosystems, cold climate, deglaciations, cyanobacteria, biological soil crust, high-altitude ecosystem, stable carbon isotope

## Abstract

Methanogens typically occur in reduced anoxic environments. However, in recent studies it has been shown that many aerated upland soils, including desert soils also host active methanogens. Here we show that soil samples from high-altitude cold deserts in the western Himalayas (Ladakh, India) produce CH_4_ after incubation as slurry under anoxic conditions at rates comparable to those of hot desert soils. Samples of matured soil from three different vegetation belts (arid, steppe, and subnival) were compared with younger soils originating from frontal and lateral moraines of receding glaciers. While methanogenic rates were higher in the samples from matured soils, CH_4_ was also produced in the samples from the recently deglaciated moraines. In both young and matured soils, those covered by a biological soil crust (biocrust) were more active than their bare counterparts. Isotopic analysis showed that in both cases CH_4_ was initially produced from H_2_/CO_2_ but later mostly from acetate. Analysis of the archaeal community in the *in situ* soil samples revealed a clear dominance of sequences related to Thaumarchaeota, while the methanogenic community comprised only a minor fraction of the archaeal community. Similar to other aerated soils, the methanogenic community was comprised almost solely of the genera *Methanosarcina* and *Methanocella*, and possibly also *Methanobacterium* in some cases. Nevertheless, ~10^3^ gdw^−1^ soil methanogens were already present in the young moraine soil together with cyanobacteria. Our results demonstrate that *Methanosarcina* and *Methanocella* not only tolerate atmospheric oxygen but are also able to survive in these harsh cold environments. Their occurrence in newly deglaciated soils shows that they are early colonizers of desert soils, similar to cyanobacteria, and may play a role in the development of desert biocrusts.

## Introduction

Methanogenic archaea are strict anaerobes and are therefore typically found in permanently water-logged, anoxic habitats such as wetlands, rice fields, marine sediments, hot springs, and the guts of ruminants and termites (Zinder, 1993; Liu and Whitman, 2008; Brune, 2011). Despite that, it has been shown that active methanogens inhabit many aerated, oxic soils including desert soils (Peters and Conrad, 1995; West and Schmidt, 2002; Angel et al., 2012). Methanogens belong to the phylum Euryarchaeota and are phylogenetically divided into six orders: Methanobacteriales, Methanococcales, Methanomicrobiales, Methanosarcinales, Methanopyrales (Ferry, 1994), and Methanocellales (Sakai et al., 2008). Recently two archaeal strains from the order Thermoplasmatales isolated from human and termite guts have been shown to be methanogenic and were reclassified as the seventh order of methanogens—the Methanoplasmatales (Dridi et al., 2012; Paul et al., 2012). While the aforementioned anoxic environments typically host a wide variety of methanogens, only the genera *Methanosarcina* and *Methanocella* were found in aerated soils, and it has been suggested that these methanogens are universal inhabitants of upland soils (Angel et al., 2012). All known methanogens possess the gene encoding for the enzyme methyl coenzyme M reductase, which is responsible for the last step of methanogenesis in all known pathways. Thanks to its universal occurrence and conserved sequence, the gene encoding for its α-subunit—the *mcrA*—is commonly used as a phylogenetic marker gene for methanogens (Lueders et al., 2001; Friedrich, 2005).

The production of biogenic CH_4_ is important in nature since it is the terminal step in the biodegradation of organic matter under anoxic conditions (Deppenmeier et al., 1996). In most terrestrial environments CH_4_ arises primarily from the reduction of CO_2_ (hydrogenotrophic methanogenesis) or the cleavage of acetate (aceticlastic methanogenesis). Although the two processes yield the same product, the source of the CH_4_ can nevertheless be differentiated by analysing the stable isotopic signatures of the carbon in the CH_4_ and its precursors (acetate and CO_2_; Conrad, 2005). The production of biogenic CH_4_ in nature typically leads to its release to the atmosphere where it acts as a greenhouse gas which is 25–33 times more potent than CO_2_ (Shindell et al., 2009).

Deserts (non-polar arid and hyperarid regions) are the largest biome on Earth, spanning over 20% of the land surface (Middleton and Thomas, 1997). Many of these deserts are characterized by a warm climate but some, particularly in high altitudes, experience frequent sub-zero temperatures throughout the year, and are termed cold deserts. The flora, fauna, and microbiota in these regions are therefore adapted to coping with low temperatures in addition to the desiccation stress common to all deserts (Dvorský et al., 2013). The area of East Ladakh, India in the western Himalayan slopes is an extensive and thinly populated high-altitude plateau characterized by both low temperatures as well as low precipitation owing to its location in the Himalayan rain shadow (Dvorský et al., 2011). This area can thus serve as a nearly pristine model for studying the ecology of cold deserts.

The soils of arid and semi-arid regions are often covered by a unique layer, a few millimeters thick and densely colonized by microorganisms, termed biological soil crust (biocrust). Biocrusts are formed by living organisms and their by-products, creating a layer of soil particles bound together by organic materials, e.g., the sticky sheath material of *Microcoleus* (Büdel, 2003). Biocrusts are predominantly composed of bacteria (cyanobacteria and others), archaea, fungi, and green and brown algae (Belnap et al., 2003; Angel and Conrad, 2013). In more humid areas, mosses, lichens, and liverworts can also be present. The particular composition of microorganisms that dominate the crust varies in nature and is largely determined by climate, soil type, the successional stage of the crust development as well as by historical contingency (Büdel, 2003; Bahl et al., 2011). In contrast to hot desert biocrusts, which are smooth and very thin, in cold climates biocrusts tend to be much thicker and develop three-dimensional structures, up to several centimeters high, in response to freeze-thaw cycles, termed as rolling and pinnacled crusts (Belnap, 2003). Whether this morphological difference is also reflected in differences in microbial community composition and function still requires research. In previous studies it has been shown that biocrusts from hot deserts can produce CH_4_ when wet (Angel et al., 2012). The primary goal of this study was therefore to investigate whether CH_4_ production can also be observed in high-altitude cold deserts such as in Eastern Ladakh. In addition, the presence of receding glaciers allowed us to also study how quickly methanogens colonize young soils. We hypothesized that similar to marine microbial mats (Hoehler et al., 2001), methanogens are an integral part of biocrusts and will be found already at early successional stages of the soil development together with cyanobacteria. Since it has been estimated that ~40% of the land area in Ladakh is covered by biocrusts (Janatková et al., 2013), we also set out to compare the potential activity and community composition between patches covered by a biocrust and bare soil.

For this purpose, soil and biocrust samples from three different vegetation belts: arid, steppe, and subnival, as well as from front and lateral moraines of receding glaciers were collected and tested for the production of CH_4_. In addition, the carbon isotopic signatures of CH_4_ and CO_2_ were analyzed to determine the pathway by which CH_4_ was formed. Finally, the methanogenic community was characterized by molecular analysis.

## Materials and methods

### Soil samples and site characterization

**Figure A1:**
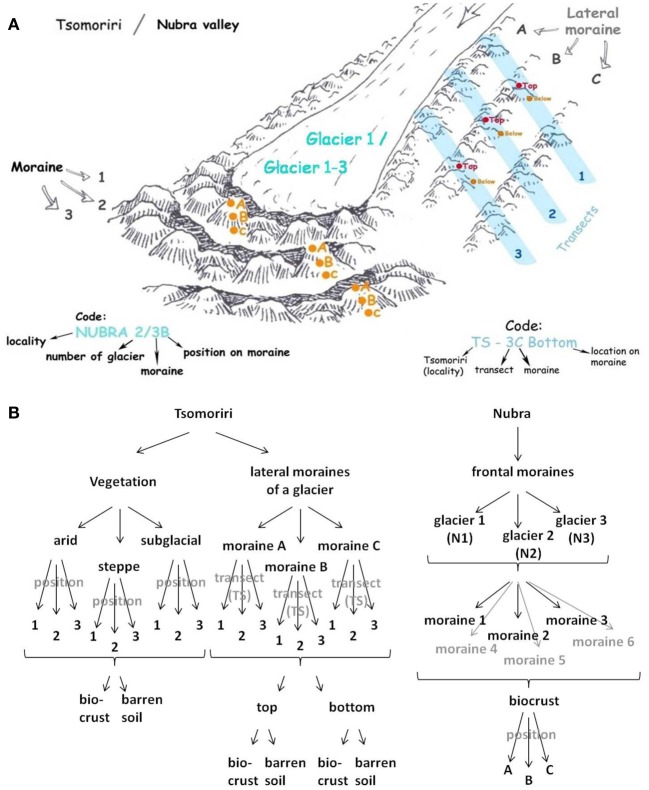
**(A)** Schematic depiction of the location of sampling sites along a glacier and code of sample denotation. **(B)** Overview of soil samples taken at different positions at Tsomoriri and Nubra. In Tsomoriri samples of matured soil were taken from three vegetation belts and of young soil from the lateral moraines of a glacier. In Nubra young soil samples were collected from the frontal moraines of three different glaciers.

Soil samples were collected from two sites in a high-altitude cold desert in Ladakh, India, from Nubra Valley and from Tsomoriri Plains. Nubra Valley is characterized by a dry climate (100 mm annual precipitation) and a neutral to alkaline soil pH. In Nubra Valley samples were collected from the frontal moraines of three glaciers (N1 – N34° 40′ 26.87″ E77° 45′ 36.17″; N2 – N34° 39′ 16.98″ E77° 45′ 8.63″ and N3 – N34° 39′ 16.10″ E77° 44′ 36.48″) located at 5400, 5300, and 5150 m above sea level, respectively. Three to six frontal moraines where studied at each glacier, and from each moraine three samples were collected at different positions (A, B, and C; Figure A1). The annual mean temperatures in these sites range from −1.6 to −3.6°C (measured at 4850 and 5250 m between Aug. 2009 and Aug. 2011; Dvorský et al., 2013). The second sampling site was Tsomoriri Plains where the annual precipitation is just under 100 mm yr^−1^ and the soil pH is neutral to alkaline. At this site, soil and crust samples from three vegetation belts were sampled: arid, steppe, and subnival located at 4700, 5300, and 5800 m, respectively (N 32°58′56,51″ E78°21′24,95″, N 32°59′31,75″, E 78°24′7,56″, N 33° 0′ 23,26″ E 78° 26′ 46,76″). Furthermore, samples from lateral moraines of the Chamser glacier (N32° 59′ 13.17″ E78° 25′ 54.084″) were also collected at altitudes 5650, 5700, and 5800 m. Here, samples were collected from lateral moraines (A, B, and C), each divided into three transects (TS1, TS2, and TS3; Figure A1). From each moraine/transect samples were collected from the top and the bottom of the moraine. The annual mean temperatures at this site range from −4.4 to −10.4°C (measured at 5350 and 5850 m between Aug. 2009 and Aug. 2011; Dvorský et al., 2013). Sample characteristics are given in Table 1. The sampling areas were partially covered by a biocrust, and soil samples were collected from either the top soil which was covered by biocrust or from bare areas (0–5 cm), which were in proximity. In Nubra Valley only biocrust samples were collected. Samples of approximately 100–150 g soil from the top layer were collected into Whirl-Pak® bags (118-ml sterile sampling bags, Nasco) and air dried in the field. Altogether a total of 84 samples were collected, and shipped at room temperature to Germany for further analysis. For nucleic acids extraction, 1 g of each sample was split in two 2 ml tubes upon arrival at the laboratory and stored at −80°C until extraction. Soil pH was determined in a 1:1 soil:water slurry solution. Soil content of total and organic carbon and total nitrogen were analyzed using an elemental analyser (vario Micro cube, CHNS mode, Elementar) by the Analytical Chemical Laboratory of the Philipps-Universität, Marburg while the stable carbon isotope signature (δ^13^C) of the total and organic carbon were analyzed at the Institute for Soil Science and Forest Nutrition (IBW) at the University of Göttingen, Göttingen, Germany, using an elemental analyser (NA2500, CE-Instruments) coupled over an interface (Conflo III, Thermo) to a mass spectrometer (Delta plus, Finnigan MAT). In both cases measurements were done before and after acidification with 10% HCl, and the difference in values was attributed to carbonate (Nüsslein et al., 2003).

**Table 1:** **Physico-chemical characteristics and methanogenic potentials of a selection of the soil samples**.

**Location**	**Site**	**Analyzed sample**	**pH**	***N*_tot_(%)^a^**	***C*_tot_(%) ^a^**	***C*_org_(%) ^a^**	**Carbonate (%)**	**Lag (*d*)**	**CH_4_ rate^b^ (nmol g^−1^ d^−1^)**
Nubra Valley	Glacier 1	N1/3A Crust	8.6	0.12 ± 0.01	2.82 ± 0.02	1.19 ± 0.03	58.0	31	169.8 ± 81.7
	Glacier 2	N2/4A Crust	8.7	0.07 ± 0.01	1.08 ± 0.08	1.02 ± 0.24	4.9	22	4.8 ± 0.5
	Glacier 3	N3/1C Crust	8.2	0.12 ± 0.01	2.46 ± 0.10	0.88 ± 0.03	64.2	13	68.4 ± 43.0
Tsomoriri Plains	Chamser Glacier	TS 3B-bottom/crust	7.5	0.24 ± 0.01	2.5 ± 0.14	2.53 ± 0.17	BLD	18	849.8 ± 143.2
		TS 3B-top/bare soil	7.5	BLD^c^	0.59 ± 0.03	0.56 ± 0.02	4.3	–	BLD
	Arid	Crust 1	8.2	0.03 ± 0.01	0.63 ± 0.07	0.28 ± 0.01	55.8	16	378.0 ± 185.6
		Bare soil 1	8.7	0.01 ± 0.00	0.16 ± 0.05	0.14 ± 0.01	13.8	21	8.9 ± 2.4
	Steppe	Crust 2	7.7	0.10 ± 0.02	0.81 ± 0.06	0.81 ± 0.00	13.5	16	21.8 ± 7.7
		Bare soil 2	8.0	0.02 ± 0.00	0.38 ± 0.04	0.25 ± 0.00	34.0	19	19.9 ± 16.8
	Subnival	Crust 3	7.0	BLD	0.96 ± 0.11	0.83 ± 0.08	0.6	16	420.1 ± 27.2
		Bare soil 3	6.9	0.02 ± 0.00	0.30 ± 0.04	0.29 ± 0.05	3.1	21	57.9 ± 13.9

a *Samples were analyzed in duplicates*.

b *Means of the 3 incubated technical replicates*.

c *BLD, below the limit of detection*.

### Incubation conditions and gas measurements

For determining the methanogenic potential of a sample, 5 g of sieved soil were incubated in a 27-ml pressure tube and amended with 5 ml sterile, distilled–deionized water in triplicates. The tubes were closed with butyl rubber stoppers (cleaned by boiling before use), purged with N_2_ and incubated at 25°C in the dark. Samples were sacrificed after CH_4_ concentration has reached about 15,000 nmol g-dry-weight^−1^ (gdw) or after 95 days if less CH_4_ was produced. Previous experience suggested that such a level of accumulated CH_4_ should allow for easy detection of methanogens through TRFLP (data not shown). Each sample was incubated in triplicated tubes representing 3 technical replicates. Prior to gas analysis, the tubes were shortly shaken by hand to equilibrate gas and aqueous phase. Measurements of CO_2_ and CH_4_ were performed following Angel et al. (2012), gas samples (500 μl) were taken from the headspace using a 500-μl glass gas-tight pressure-lock syringe (Vici) every 7–14 days and analyzed immediately. Methane and CO_2_ concentrations were analyzed using a GC (GC-8A; Shimadzu) equipped a 3 m, ø 1/8″ stainless steel column filled with Hayesep Q 80/100 mesh, a methanizer (Ni-catalyst at 350°C, Chrompack) and a flame ionization detector (SRI, temperature: 160°C). The injector temperature was 160°C, the oven temperature: 120°C, and the carrier gas was H_2_.

The isotopic signatures of the carbon in the CH_4_ and CO_2_ were determined using a Gas Chromatograph Combustion Isotope Ratio Mass Spectrometer (GC-C-IRMS; Trace GC Ultra, Thermo Fischer Scientific), following Conrad et al. (2009). The principle operation of GC-C-IRMS has been described by Brand (1996). The CH_4_ and CO_2_ in a 500 μ l gas samples were first separated in a Trace GC Ultra Gas Chromatograph using a Pora PLOT Q column (27.5 m length, 0.32 mm i.d.; 10 μm film thickness; Varian, Palo Alto, CA, USA) at 30°C with helium (99.996% purity; 2.6 ml/min) as carrier gas. After conversion of CH_4_ to CO_2_ in a GC Isolink 1030 Oxidation Reactor at 940°C, the isotope ratio of ^13^C/^12^C was analyzed in an IRMS (Delta V Advantage, Thermo). The isotope reference gas was CO_2_ (99.998% purity; Air Liquide), calibrated with the working standard methylstearate (Merck). Values are reported in the delta notation against the Vienna Pee Dee Belemnite (Hayes, 1993):

(1) $$\delta^{13}\text{C}=10^{3}(R_{\text{sa}}/R_{\text{st}}-1)$$

Where *R*_sa_ = ^13^C_sa_/^12^C_sa_ and *R*_st_ = ^13^C_st_/^12^C_st_ of sample (sa) and standard (st), respectively.

The preference of a reaction for the light carbon isotope is described by the fractionation factor (α), which is defined as:

(2) $$\alpha =\left(\delta_{\text{Substrat}}+1000\right)\text{​}/\text{​}\left(\delta_{\text{Product}}+1000\right)$$

equivalent to:

(3) ε=(1−α)1000

Where δ_Substrat_ and δ_Product_ are the δ^13^C values of the substrate and the product, respectively.

The relative fraction of the hydrogenotrophically derived CH_4_ was calculated with the following equation:

(4) $$f_{\text{H2}}=(\delta^{13}{\text{C}}_{\text{CH4}}-\delta_{\text{ma}})/(\delta_{\text{mc}}-\delta_{\text{ma}})$$

Where δ^13^C_CH4_ = δ^13^C of CH_4_ in the headspace; δ_ma_ is the δ^13^C of CH_4_ produced solely from acetate and δ_mc_ is the δ^13^C of CH_4_ produced solely from H_2_/CO_2_. Values of δ_ma_ and δ_mc_ were estimated from measured δ^13^C_CO2_ (δ^13^C of CO_2_ in the headspace) using fractionation factors (α or ε values) from the literature and assuming that the δ^13^C of acetate (δ^13^C_ac_) is identical to the δ^13^C of organic matter (δ^13^C_org_):

(5) $$\delta_{\text{ma}}=\delta^{13}{\text{C}}_{\text{org}}+\varepsilon_{\text{acetate},\text{CH4}}$$

(6) $$\delta_{\text{mc}}=\delta_{\text{CO2}}+\varepsilon_{\text{CO2},\text{CH4}}$$

### Extraction and amplification of nucleic acids

After incubations were completed, the pressure tubes were opened and the slurries were centrifuged at 10,000 rpm for 2 min to remove pore water. One gram of soil slurry was frozen in liquid nitrogen and stored at −80°C. Total nucleic acids were extracted from 0.5 g of the dry unincubated soil as well as from the incubated slurry samples as previously described (Angel, 2012; Angel et al., 2012).

The primers for the PCR reactions are listed in Table 2. Each PCR reaction was 50 μl in volume and contained: 10 μl GoTaq®Flexi 5× Green Buffer (Promega), 0.2 mM dNTP mixture, 1.5 mM MgCl_2_, 0.8 μg/μl BSA (Roche), 0.25 μM of each primer, 1.5 U of GoTaq® DNA polymerase (Promega), and 1 μl of DNA template. The following programme was used: 94°C for 4 min followed by 30 cycles of 94°C for 30 s, 52°C for 30 s, and 72°C for 45 s and a single step of final elongation at 72°C for 10 min.

**Table 2:** **Overview of the used primers in this study**.

**Oligo. name^a^**	**Target**	**Oligo. sequence (5'–3')**	**Position^b^**	**GC (%)**	**Tm**	**Amplicon size**	**Assay**	**References**
ARC 109-F	Archaea 16S rRNA gene	ACK GCT CAG TAA CAC GT	109–125	47	54	826	TRFLP sequencing	Großkopf et al., 1998
ARC 934-R		GTG CTC CCC CGC CAA TTC CT	915–934	65	71			
CYA 359_mod-F	Cyanobacteria 16S rRNA gene	GRG GAA TYT TCC GCA ATG GG	359–378	60	63	447	qPCR	Nuebel et al., 1997
CYA 781-R		GAC TAC WGG GGT ATC TAA TCC CWT T	791–805	46	64			
mlas-mod-F	Universal *mcrA* gene	GGY GGT GTM GGD TTC ACM CAR TA	976–998a	43–65	68	469	qPCR	Steinberg and Regan, 2008
mcrA-rev-R		CGT TCA TBG CGT AGT TVG GRT AGT	1421–1444a	42–54	66			

a *F, forward primer; R, reverse primer*.

b *Positions are based on the following: primers targeting the 16S rRNA gene—E.coli; primers targeting the mcrA gene—M. thermautotrophicus mcrA gene accession number: U10036 (following Steinberg and Regan, 2008)*.

For analysis of terminal restriction fragment length polymorphism (TRFLP) of archaeal 16S rRNA genes, the forward primer 109f was labeled with 6-FAM (6-carboxyfluorescein) at the 5′ end. Approximately 200 ng of purified PCR products were digested overnight at 65°C with the restriction enzyme Taq^α^ 1 (New England BioLabs). TRFLP sample preparation and processing were performed as previously described (Angel et al., 2012). For analysis, the height of the measured peaks was used, and expressed in relative abundance of the total height. All peaks under 1% of the total height were treated as noise and were removed.

### Cloning and sequencing and phylogenetic analysis

Four samples (TS 2B-top/biocrust, TS 3B-bottom/biocrust, subnival-biocrust 3, and subnival-bare soil 2) showing the most diverse TRFLP patterns were used for cloning of archaeal 16S rRNA genes and Sanger sequencing, in order to assign TRFs to genus. Libraries were constructed using purified PCR products (GenElute PCR cleanup kit, Sigma) which were cloned into the pGEM-T easy vector (Promega) following the manufacturer's instructions. Sanger sequencing services were provided by GATC (Germany) and M13f and M13r primers were used to sequence from both flanking regions of the vector to the insert. Twenty four clones from each sample were analyzed (96 clones in total). Only reads that fulfilled the following criteria were used: the two reads were overlapping up to the opposite primer sequence, the assembled contig was longer than 800 bp, the contig was confirmed to be archaeal and also not chimeric using BLAST. Sixty six sequences passed our quality filtering and were used for phylogenetic analysis. Sequences were aligned against the SILVA 108 SSU Ref database (Quast et al., 2013) using the ARB software package (Ludwig et al., 2004). The phylogenetic tree was calculated using RAxML (Stamatakis, 2006), implemented in Arb, using rapid hill climbing algorithm, PROTMIXJTT evolutionary model and a 1000 bootstrap runs. Nucleotide sequences were submitted to GenBank and can be found under the following accession numbers: KF445438–KF445503.

### Quantitative real-time PCR assays

Quantitative real-time PCR (qPCR) assays were used to quantify the 16S rRNA gene of cyanobacteria and the *mcrA* gene of methanogens. The assays were based on SYBR® Green and were performed as previously described (Angel et al., 2011; Angel and Conrad, 2013). A standard containing a known number of DNA copies of the target gene was used for all assays in serial dilutions for generating a calibration curves. These standards were generated from an environmental clone of a cyanobacterium and from a pure culture of *Methanosarcina thermophila*. All qPCR reactions were performed on an iCycler thermocycler equipped with MyiQ™ detection system (BioRad). The resulting data were analyzed with the iQ5 optical systems software (BioRad). Accounting for the dilutions of DNA template, our assays allowed detecting 5 × 10^2^ copies per gram dry soil.

## Results

### Soil characteristics

The pH in the samples obtained from the vegetation belts and the lateral moraines (Tsomoriri) was neutral to alkaline (pH 6.93–8.66), which is typical for desert soils, while the samples from the frontal moraines of Nubra Valley were slightly more alkaline (pH 8.14–8.96). Overall the samples were poor in both carbon and nitrogen. Total carbon content ranged from 0.16 to 2.8% while that of nitrogen was below the detection limit in many samples and reached at most 0.24% of the dry soil mass (Table 1). Further analysis showed that in many of the samples much of the carbon was inorganic, i.e., carbonate, which made up as much as ~60% of the total carbon in the samples (Table 1). The isotopic signature of the carbon in the soil typically ranged from −10 to −22‰ and tended to be heavier in samples with higher carbonate content (Table 3).

**Table 3:** **Stable carbon isotope analysis**.

**Location**	**Site**	**Analyzed sample**	**δ13C_tot_(‰)**	**δ13C_org_(‰)**	**δ13C_CH4_Begin^a^^,^^b^**	**δ13C_CH4_End**	**δ13C_CO2_Begin^a^^,^^b^**	**δ13C_CO2_End**	**f_H2_Begin^a^^,^^b^**	**f_H2_End**
Nubra Valley	Glacier 1	N1/3A Crust	−10.3	−23.6	−71	−38.7	−18.4	−20.7	0.7–1.0	0.1–0.2
	Glacier 2	N2/4A Crust	−20.5	−25.2	−66.4	−50.4	−24.1	−24.1	0.6–0.9	0.3–0.5
	Glacier 3	N3/1C Crust	−10.5	−23.1	−58.2	−53.8	−21.7	−20.4	0.4–0.7	0.4–0.6
Tsomoriri	Chamser	TS 3B-bottom/crust	−22.2	−22.4	−64.1	−39.8	−14.6	−19.1	0.5–0.8	0.1–0.2
Plains	Glacier	TS 3B-top/bare soil	−20.9	−21.2	BLD^c^	BLD	BLD	BLD	BLD	BLD
	Arid	Crust 1	−14.0	−19.7	−52.7	−47.4	−11.9	−14.6	0.4–0.5	0.3–0.4
		Bare soil 1	−13.6	−24.0	−61	−64.2	−24.1	−22	0.5–0.8	0.6–0.8
	Steppe	Crust 2	−17.5	−20.4	−69.7	−70	−16.5	−19.7	0.6–1.0	0.6–1.0
		Bare soil2	−22.3	−23.9	−69.6	−52.4	−23.6	−22.8	0.6–1.0	0.3–0.5
	Subnival	Crust 3	−21.6	−21.3	−69.8	−43.5	−4.4	−12.4	0.6–1.0	0.2–0.3
		Bare soil 3	−22.3	−22.1	−62.6	−41.4	−27.0	−22.4	0.5–0.8	ca. 0.2

a *Means of the 3 technical replicates*.

b *“Begin” and “end” refer to the first and last measurements*.

c *BLD, below the limit of detection*.

### Methane production potential and the stable isotope ratio of CH_4_ and CO_2_

Methane production was tested in all 84 samples. The CH_4_ production rate was always higher in the biocrust samples than in the bare soil samples. In total, 50 (60%) of the samples produced at least some CH_4_ (>1 nmol gdw^−1^ d^−1^; Table 1; Table A1). The CH_4_ production rate of 14 highly methanogenic samples (~30% of the methanogenic samples) reached over 100 (and in some cases up to around 1000) nmol gdw^−1^ d^−1^. Linear CH_4_ accumulation rates were detected in the vegetation belt samples at Tsomoriri Plains after a lag phase of 19 days on average (Table 1; Table A1). First traces of CH_4_ were detected in the lateral moraine samples at Tsomoriri Plains after a lag phase of 24 days on average and in Nubra Valley samples after 31 days on average.

All samples from the different vegetation belts produced CH_4_ (Table 1; Table A1). The highest methanogenic activity was found in the subnival soil samples with 835 ± 228 and 65 ± 5 nmol CH_4_ gdw^−1^ d^−1^ on average in the crust and bare soil, respectively, followed by the arid samples with 329 ± 124 and 19 ± 14 nmol CH_4_ gdw^−1^ d^−1^ on average in the crust and bare soil, respectively and by the steppe samples with only 99 ± 69 and 17 ± 6 nmol CH_4_ gdw^−1^ d^−1^ on average in the crust and bare soil, respectively.

Also the soils from the lateral moraines of the receding Chamser glacier in Tsomoriri Plains showed methanogenic potential, though not in all samples and in lower amounts compared to the vegetation belts samples. In transect 1 samples did not produce CH_4_, in transect 2, only 4 of the 10 samples produced CH_4_, and in the last transect (3), 9 of the 10 samples produced CH_4_. Here as well, biocrust samples produced more CH_4_ than the bare soils in nearly all cases except moraine C, where the bare soil sample produced slightly more CH_4_ than the sample with the crust (Table A1). On average, the active biocrust samples produced CH_4_ at a rate of 239 ± 114 nmol CH_4_ gdw^−1^ d^−1^ while the active bare soil samples produced only 89 ± 48 nmol CH_4 gdw^−1^ d^−1^._

In Nubra Valley a larger proportion of the samples were active compared to the Tsomoriri Chamser glacier samples and all three glacier moraines showed at least some CH_4_ production, but most samples produced very little CH_4_. On average, active samples here (which were all biocrust) produced 20 ± 8 nmol CH_4_ gdw^−1^ d^−1^.

**Figure 1:**
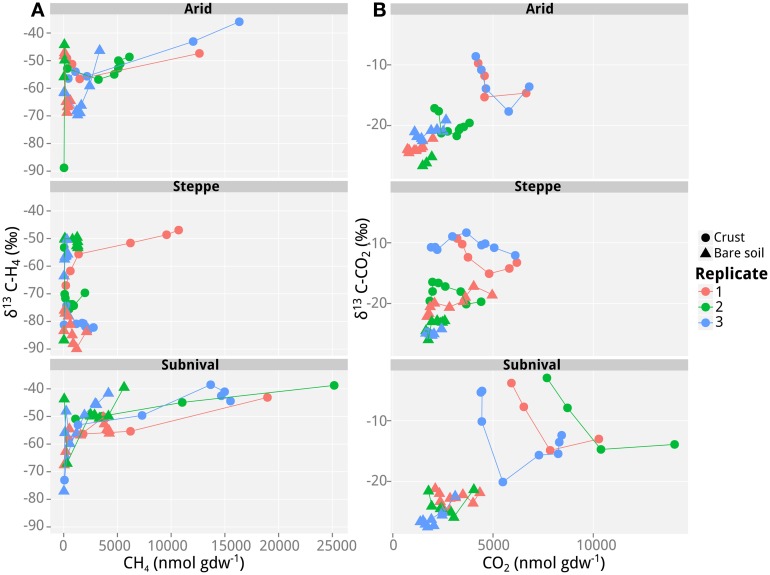
**Values of δ^13^C of CH_4_ and CO_2_ as a function of CH_4_ and CO_2_ concentrations in incubations with soil samples from the different vegetation belts in Tsomoriri Plains**. Depicted are the three vegetation belts samples: arid, steppe, and subnival. **(A)** δ^13^C of CH_4_ and **(B)** δ^13^C of CO_2_. Only samples producing >40 nmol g^−1^ CH_4_ produced reliable δ^13^C measurements of CH_4_ and are shown here. Temporal progression is always along the x-axis. Three biological replicates were analyzed and each data point represents a mean of *n* = 3 technical replicates.

**Figure 2:**
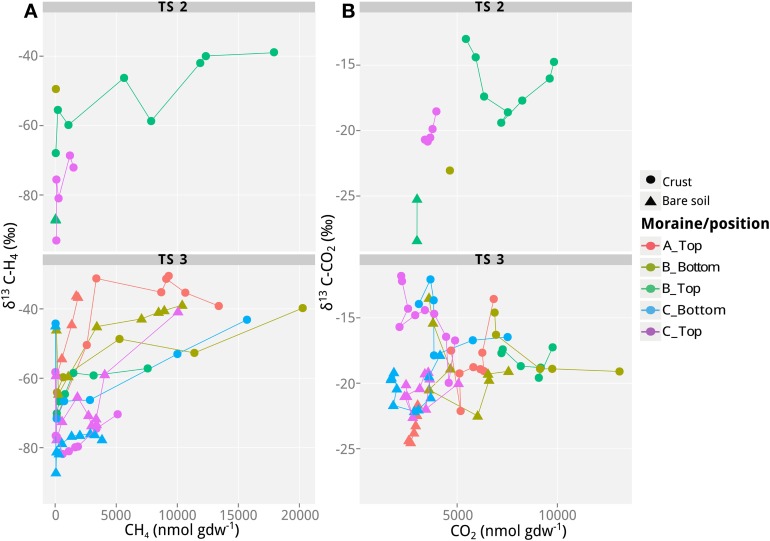
**Values of δ^13^C of CH_4_ and CO_2_ as a function of CH_4_ and CO_2_ concentrations in incubations with soil samples from the different lateral moraines in Tsomoriri Plains. (A)** δ^13^C of CH_4_ and **(B)** δ^13^C of CO_2_. Only samples producing >40 nmol g^−1^ CH_4_ produced reliable δ^13^C measurements of CH_4_ and are shown here. Therefore, only transects TS2 and TS3 are plotted. Temporal progression is always along the x-axis. Means of *n* = 3 (technical replicates).

**Figure 3:**
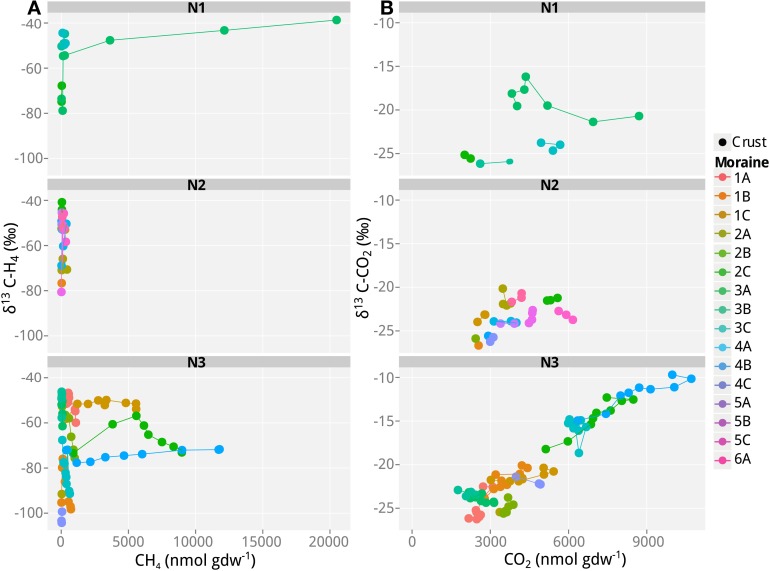
**Values of δ^13^C of CH_4_ and CO_2_ as a function of CH_4_ and CO_2_ concentrations in incubations with soil samples from the different frontal moraines in Nubra Valley. (A)** δ^13^C of CH_4_ and **(B)** δ^13^C of CO_2_. Only samples producing >40 nmol g^−1^ CH_4_ produced reliable δ^13^C measurements of CH_4_ and are shown here. N1, glacier 1 in Nubra Valley, containing 4 moraines; N2, glacier 2 with 6 moraines, N3, glacier 3 with 4 moraines. Temporal progression is always along the x-axis. Means of *n* = 3 (technical replicates).

In addition to the measurement of gas concentrations, the isotopic signatures of the carbon containing compounds, namely CH_4_ and CO_2_, were measured in this study. The isotopic signatures (δ^13^C) of the carbon in the CH_4_ and CO_2_ indicate which methanogenic pathway—hydrogenotrophic or aceticlastic—was favored in our incubations. The δ^13^C of the CH_4_ ranged from −100 to −30‰, and showed generally a positive trend with time (and therefore also with concentration; Figures 1–3). At the beginning of the incubations, the average δ^13^C_CH4_ values of all analyzed samples were around −70 to −52‰ (Figures 1–3). Over time values shifted to heavier δ^13^C in the soil samples from the lateral (δ^13^C_CH4_ = −40‰; Figure 2) and the frontal moraines (δ^13^C_CH4_ = −54 to −32‰; Figure 3). In contrast, the isotopic signature of CH_4_ of the steppe-biocrust sample as well as the bare soil samples from arid sites were relatively constant over time (δ^13^C_CH4_ = −70 to 64‰; Figure 1). As for CO_2_, the δ^13^C ranged from −30 to −5‰ (Figures 1–3). The bare soil samples showed a heavier isotopic signature in the CO_2_ compared with the biocrust samples.

The characteristic δ^13^C values of CH_4_ and CO_2_ at the beginning and the end of incubation are summarized in Table 3. These values were used to calculate the fraction of hydrogenotrophic methanogenesis (*f*_H2_) using Equation (4). For these calculations we assumed ε_ac,CH4_ = −10‰ (Goevert and Conrad, 2009; Angel et al., 2012) and δ^13^C_ac_ = δ^13^C_org_. On average, δ_ma_ was therefore −32.2‰. We further assumed previously published fractionation factors for conversion of H_2_/CO_2_ to CH_4_ (−73 and −49‰; Fey et al., 2004; Conrad et al., 2010) so that δ_mc_ was in the range of −90 to −70‰. The relative fraction of CH_4_ derived from H_2_/CO_2_ in the early emitted methane was relatively high (*f*_*H*2_> 0.50 in almost all samples; Table 3). Thereby the samples seem to be generally dominated by hydrogenotrophic methanogenesis in the beginning. Toward the end of the incubation, however, *f*_*H*2_ dropped to 0.10–<0.50. Nevertheless, in some samples (arid-bare soil, steppe-biocrust, and glacier 3–1C biocrust) hydrogenotrophically derived CH_4_ remained a dominant pathway over time (*f*_*H*2_ = 0.4–1.0).

### Composition of the archaeal community

**Figure 4:**
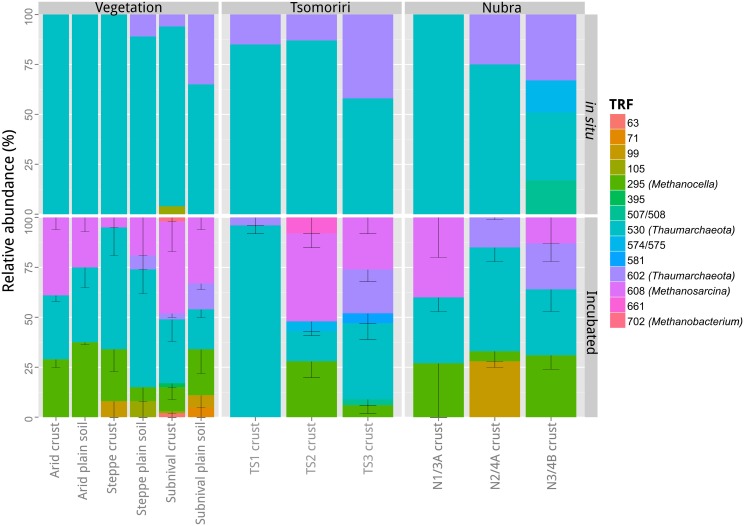
**TRFLP community profiles of archaeal 16S rRNA genes from different soil samples**. The community composition was characterized in a selection of *in situ* and incubated soil samples from the three vegetation belts, the lateral moraines in Tsomoriri Plains and the frontal moraines in Nubra Valley. For the incubated samples technical replicates were analyzed. Means ± SE of *n* = 2 (technical replicates). TS1, Tsomoriri moraine 1; TS2, Tsomoriri moraine 2; TS3, Tsomoriri moraine 3; N1/3A, Nubra glacier 1 moraine 3a; N2/4A, Nubra glacier 2 moraine 4a; N3/4B, Nubra glacier 3 moraine 4b.

**Figure A2:**
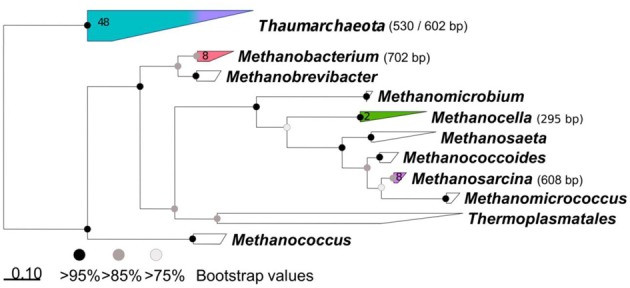
**Archaeal 16S rRNA phylogenetic tree**. Maximum likelihood phylogenetic tree based on aligned partial 16S rRNA gene sequences obtained from clones from four incubated soil samples. Clusters containing sequences obtained in this study are shown in colors. The length of the terminal restriction fragment is indicated in brackets. The number of sequences obtained is indicated in each cluster. Scale bar: substitutions per base.

TRFLP was used to characterize the archaeal community in different samples. TRFLP profiles were generated only for a subset of the total samples, i.e., for one original sample (before incubation) from each vegetation belt and glacial moraine at Tsomoriri or Nubra, and for two out of three of the respective incubation technical replicates. The tested samples showed similar occurrence of TRFs but their proportion varied between sites (Figure 4). Altogether some 14 different TRFs could be reliably detected in the analyzed samples, but only five of the major TRFs could be identified in the sequences from the clone library (Figure A2).

The diversity of archaea in the *in situ* samples was dominated (80–100% of TRFs) by Thaumarchaeota (TRFs 530 and 602 bp) while no methanogens could be detected. Following incubation, the diversity of archaea increased with the appearance of methanogens. *Methanosarcina* (608 TRF) and *Methanocella* (295 TRF) appeared in all CH_4_ producing samples and in one case also *Methanobacterium* (702 TRF) was detected. The proportion of methanogens in the incubated methanogenic samples increased to 30–100% of the total community, with the level of enrichment correlating with the methanogenic potential. The nine TRFs (63, 71, 99, 105, 395, 507, 574, 581, 661 bp) which could not be affiliated to the sequence data occurred only occasionally in the samples, and made up only ~14% of the population. In the case of one sample from Nubra Valley (N2/4A), unidentified TRF = 99 bp was shown to contribute 27% to total T-RFs. However, the CH_4_ production rate in this sample was very low, thus this TRF is unlikely to have represented a methanogen.

### Quantification of cyanobacteria and methanogens along a lateral moraine

**Figure 5:**
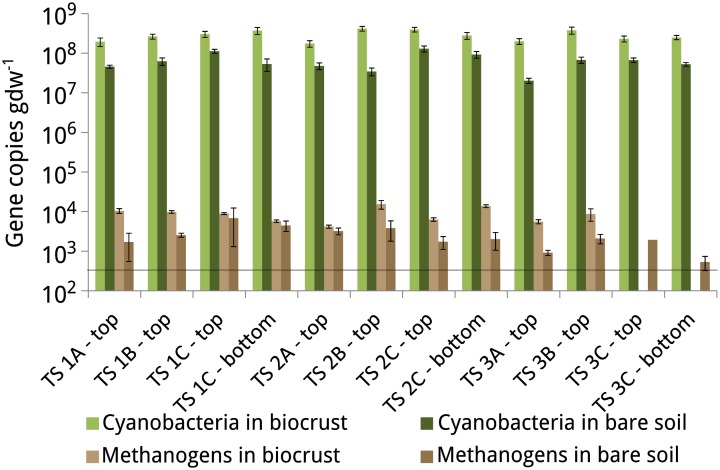
**Gene copy numbers of the 16S rRNA gene of cyanobacteria and the *mcrA* gene of methanogens in the biocrusts and bare soils of the lateral moraines in Tsomoriri Plains**. Horizontal line: detection limit (5 × 10^2^ gene copies gdw^−1^). Means ± SE of *n* = 6 (2 independent qPCR reactions containing 3 replicates of the PCR mixture).

To determine how quickly methanogens colonize soils that were newly exposed by deglaciation and to better understand the relationship between methanogens and biocrusts, we quantified the density of methanogens and cyanobacteria in the soil samples obtained from the lateral moraines at Tsomoriri-Plains. Cell densities of methanogens (as determined from the *mcrA* gene copies) were close to the detection limit of 5 × 10^2^ gene copies per gram dry weight and ranged from 5 × 10^2^ to 1.5 × 10^4^ (Figure 5). The cell densities of cyanobacteria (estimated from the 16S rRNA gene copy numbers) were much higher and ranged from 2 × 10^7^ to 4.2 × 10^8^. The cell densities of both cyanobacteria and methanogens were relatively constant with respect to the glacier moraine, with no apparent pattern. However, in all cases the cell densities of both cyanobacteria and methanogens were higher in biocrust samples compared to bare soil samples.

## Discussion

The occurrence of methanogens in dry soils of hot deserts has previously been demonstrated (Angel et al., 2011, 2012). Here we tested for their presence in high-altitude cold deserts, in both mature as well as in very young soils, recently exposed by glacial recession. East Ladakh in the north of India is characterized by both very high altitudes (leading to a very cold climate) as well as little precipitation, resulting from its geographical location in the monsoon rain shadow. These characteristics together with its pristine nature made it an ideal location for performing these tests.

Overall, all three sites examined in this study (Nubra Valley glaciers, Tsomoriri Plains glaciers, and vegetation belts at Tsomoriri Plains) showed methanogenic potential, though not in all samples and to varying degrees. As expected, only some of the samples from the younger moraine soils produced CH_4_ in our incubations, and the production rates were nearly always lower than in the samples from the various vegetation belts, where the soil is more mature. The production of endogenous CH_4_ under anoxic slurry conditions in soils obtained from high altitudes stands in contrast to a previous report by West and Schmidt (2002), who could stimulate methanogenesis in alpine soils only by supplying H_2_ to their slurries. Hence, it seems that methanogens were present in their soils, but were limited by the supply of substrate. In the Himalayan samples, on the other hand, the entire microbial cascade involved in degradation of organic matter to CH_4_ plus CO_2_ was apparently present and could be activated upon anoxic incubation.

The vegetation belt samples produced CH_4_ at rates reaching up to 1200 nmol gdw^−1^ d^−1^ while the moraine soil samples reached at most 850 and 142 nmol gdw^−1^ d^−1^ CH_4_ for the lateral moraines from Tsomoriri Plains and the frontal moraines in Nubra Valley, respectively. These rates (particularly for the vegetation belt samples) are well within the range of values previously reported by Angel et al. (2012) for upland soils from various parts the world under the same incubation conditions, indicating little effect of the particular local stress factors (e.g., low temperatures) on the potential methanogenic activity of the soils. Also the fact that of the three vegetation belts tested, the subnival one, which is the coldest but the wettest one, showed the highest methanogenic rates indicates that even in this cold-desert, water scarcity is more limiting than temperature. Similar to previous reports by Angel et al. (2012), biocrust samples always exhibited higher CH_4_ production rates than their bare soil counterparts.

Analysis of the soil organic matter and nitrogen contents in the samples showed very low values and overall little difference between sites (although they were generally lower in the Tsomoriri vegetation belts samples). Lack of substrate can therefore not explain the difference in CH_4_ production rates between the samples and the lack of methanogenic potential in many of the moraine soils. Instead we suggest that it is the development of the microbial community (and particularly the development of mature biocrusts) that plays a crucial role in determining whether CH_4_ will be produced or not and to what extent, i.e., how much time has passed from the glacier recession. The observed CH_4_ production gradient along the lateral moraines of Tsomoriri Plains and the frontal moraines of Nubra Valley would sustain this theory because the oldest soils (TS3/N3) showed the highest rates and the greatest proportion of active methanogenic samples. Nemergut et al. (2007) who studied the development of microbial communities along a receding glacier in Peru reported not just an increase in the complexity of the microbial communities with soil age but also an overall increase in similarity between the communities in the young soil to that of a mature soil with age. Similarly, Gangwar et al. (2009) could show an increase in both bacterial population size and diversity with increasing altitude in Western Himalayian mountain tops. A similar trend was reported for the Rocky Mountains (Bryant et al., 2008). This indicates that despite being considered “dispersion unlimited” (Lindström and Langenheder, 2012) soil bacterial communities, much like plants, undergo successional development over time and do not simply grow in size from the moment of soil exposure.

Analysis of the stable isotopes signatures as a reflection of the pathways leading to the formation of CH_4_ showed a clear dominance of the hydrogenotrophic pathway (~50–100%) during the first third of the incubation period, but a shift toward the aceticlastic pathway in the latter part of the incubation. While this is a typical pattern for such slurry incubations and has often been reported in the past, also for upland soils (e.g., Roy et al., 1997; Glissmann and Conrad, 2002; Angel et al., 2012), this might not reflect well the processes as they occur in the field, particularly for aerated soils. Angel et al. (2012) showed that in the presence of oxygen acetate is consumed by other processes (probably heterotrophic ones) and CH_4_ is produced exclusively from H_2_/CO_2_.

Initiation of CH_4_ production commenced after a lag of 19–34 days on average. The lag time for the mature soils from the various vegetation belts was in agreement with lag times measured for other upland soils, but the moraine soils needed significantly more time to initiate CH_4_ production (Angel et al., 2012). Assuming no inhibitors in the soil and accounting for the very low concentrations of alternative electron acceptors such as NO^3−^ (>2 mg kg^−1^; Janatková et al., 2013) we assumed that the long lag times resulted from the low population size of the methanogens. Indeed, the initial population density of methanogens in the soil from Tsomoriri moraines was very low (10^2^–10^4^ copies gdw^−1^) and in several cases even below the detection limit. However, it was about tenfold larger in the biocrusts, showing again that biocrusts are favorable habitats for methanogens despite their photosynthetic nature. Still, these values are lower than those obtained for mature biocrusts from the Negev Desert (Angel et al., 2012), indicating either that these Himalayan crusts still undergo development, or that cold desert crust inherently harbor a smaller methanogenic community. The numbers of methanogens were also much lower than the densities observed in anoxic soils such as rice fields (usually 10^6^ to 10^7^ copies gdw^−1^; Conrad and Frenzel, 2002).

Cyanobacteria are typically the dominant primary producers in biocrusts and also act as the pioneer colonizers in the successional development of the crust (Büdel, 2003). In a previous study it was shown that cyanobacteria comprised 70–99% of all phototrophs in these Himalayan slopes, and were particularly dominant in the newly exposed moraines (Řeháková et al., 2011; Janatková et al., 2013). Also our quantification of cyanobacteria in the samples from Tsomoriri Plains showed a high density of these organisms even in these young soils. Nevertheless, numbers of cyanobacteria were lower in the bare soil samples than the biocrusts and were overall lower by about an order of magnitude compared to mature crusts from hot deserts (Steven et al., 2012; Angel and Conrad, 2013). The association between cyanobacteria and methanogens is well-known in marine mats (Hoehler et al., 2001) and has also been postulated for desert crusts (Angel et al., 2011). This notion is strengthened here with the occurrence of methanogens in these newly developed crusts alongside cyanobacteria, which are typical for mature crusts (e.g., lichens and mosses; Belnap and Eldridge, 2003). As the main nitrogen fixers in arid environments (Evans and Ehleringer, 1993) cyanobacteria are crucial for the proliferation of nearly all other microorganisms in the soil, including methanogens. Brankatschk et al. (2011) pointed out that N_2_ fixation, mineralization, nitrification and denitrification are important drivers of N turnover in young soils and that soils along a glacier forefield were characterized by a high abundance of N_2_ fixing organisms. Also Nemergut et al. (2007) showed that cyanobacteria played an important role in the soil development of unvegetated, recently deglaciated soil and that the cyanobacteria were the abundant N_2_-fixing clade. Apart from supplying nitrogen it is also possible that methanogens in biocrusts rely on cyanobacteria for the supply of degradable organic matter or even hydrogen, as is known to occur in hypersaline mats (Hoehler et al., 2001).

Fingerprinting of the archaeal community in the native soils showed that they were dominated by Thaumarchaeota, which is common for upland soils (formerly considered to be Crenarchaeota; Bates et al., 2011), and even for desert biocrusts specifically (Soule et al., 2009; Angel et al., 2012). Only few members of the thaumarchaeotal phylum have been cultivated so far, all of which seem to belong to the ammonia oxidizing archaea guild (Offre et al., 2013). Yet considering the abundance of Thaumarchaeota in natural environments it cannot be excluded that they perform other biogeochemical processes than ammonia oxidation. The dominance of Thaumarchaeota indicates that the methanogenic population comprised less than 1% of the initial archaeal population (the typical detection limit of TRFLP). In fact, considering our quantitative measurements of ~10^3^ methanogens gdw^−1^ and an average population size of ~10^7–10^8^^ copies gdw^−1^ for archaea in desert biocrusts (Soule et al., 2009; Angel and Conrad, 2013) methanogens most likely comprised less than 0.01% of the archaeal population. This estimation of total archaeal population size is also supported by recent quantification of soils from this environment (RA, unpublished). Similar to previous reports on methanogens from upland soils (both arid and humid), this high-altitude cold desert was also dominated by methanogens of the genus *Methanosarcina* and *Methanocella* (Nicol et al., 2003; Poplawski et al., 2007; Angel et al., 2012; Scavino et al., 2013). This is in stark contrast to the communities found in typical methanogenic environments, such as wetlands, rice fields, lake sediments, and guts of ruminants or termites, which host a variety methanogenic genera simultaneously (Lueders et al., 2001; Sjoeling and Cowan, 2003; Banning et al., 2005; Brune, 2011; Nicholson et al., 2007). It has been postulated that the occurrence of only these two genera of methanogens is related to their relative oxygen tolerance compared to other methanogens (Erkel et al., 2006; Angel et al., 2011), and the findings of this study further corroborate the notion that *Methanosarcina* and *Methanocella* are globally distributed upland soil methanogens. However, the environment in these Himalayan mountaintops poses yet another type of stress in the form of extreme cold temperatures and frequent freeze-thaw cycles. It is therefore not surprising that two previous studies found a majority of psychrophilic and psychrotolerant bacteria in the communities from these soils (Nemergut et al., 2007; Gangwar et al., 2009). Hence, *Methanosarcina* and *Methanocella* appear to be also psychrotolerant in addition to their ability to tolerate air exposure and desiccation.

### Conflict of interest statement

The authors declare that the research was conducted in the absence of any commercial or financial relationships that could be construed as a potential conflict of interest.

## Appendix

**Table A1:** **Methane production rates and lag times of all samples**.

**Vegetation belts**	**CH_4_-rate (nmol g^−1^ d^−1^)^a^**	**Lag (*d*)**	**Nubra Valley^b^**	**CH_4_-rate (nmol g^−1^ d^−1^)**	**Lag (*d*)**	**Tsomoriri Plains**	**CH_4_-rate (nmol g^−1^ d^−1^)**	**Lag (d)**
Arid-biocrust 1	378.0	16	N1/1A	BLD^c^		TS 1A-top/biocrust	BLD	
Arid-biocrust 2	93.0	14	N1/1B	BLD		TS 1A-top/bare soil	BLD	
Arid-biocrust 3	515.9	17	N1/1C	BLD		TS 1A-bottom/biocrust	BLD	
Arid-bare soil 1	8.9	21	N1/2A	BLD		TS 1A-bottom/bare soil	BLD	
Arid-bare soil 2	2.7	41	N1/2B	BLD		TS 1B-top/biocrust	BLD	
Arid-bare soil 3	46.5	22	N1/2C	0.51	43	TS 1B-top/bare soil	BLD	
Steppe-biocrust 1	236.1	16	N1/3A	169.8	31	TS 1C-top/biocrust	BLD	
Steppe-biocrust 2	21.8	16	N1/3B	2.3	32	TS 1C-top/bare soil	BLD	
Steppe-biocrust 3	38.2	12	N1/3C	3.0	31	TS 1C-bottom/biocrust	BLD	
Steppe-bare soil 1	25.2	19	N2/1A	BLD		TS 1C-bottom/bare soil	BLD	
Steppe-bare soil 2	19.9	19	N2/1B	0.4	38	TS 2A-top/biocrust	BLD	
Steppe-bare soil 3	6.4	25	N2/1C	2.4	41	TS 2A-top/bare soil	BLD	
Subnival-biocrust 1	875.9	14	N2/2A	5.2	47	TS 2B-top/biocrust	330.8	32
Subnival-biocrust 2	1208.0	14	N2/2B	0.3	45	TS 2B-top/bare soil	0.3	39
Subnival-biocrust 3	420.1	16	N2/2C	1.42	18	TS 2B-bottom/biocrust	BLD	
Subnival-bare soil 1	125.6	16	N2/3A	BLD		TS 2B-bottom/bare soil	BLD	
Subnival-bare soil 2	75.8	20	N2/3B	BLD		TS 2C-top/biocrust	15.7	33
Subnival-bare soil 3	57.9	21	N2/4A	4.8	22	TS 2-top/bare soil	BLD	
			N2/4B	0.3	41	TS 2C-bottom/biocrust	0.1	
			N2/4C	1.1	40	TS 2C-bottom/bare soil	BLD	
			N2/5A	0.8	48	TS 3A-top/biocrust	62.9	14
			N2/5B	1.8	41	TS 3A-top/bare soil	10.0	28
			N2/5C	5.4	26	TS 3B-top/biocrust	87.6	35
			N2/6A	2.1	21	TS 3B-top/bare soil	BLD	
			N3/1A	11.27	23	TS 3B-bottom/biocrust	849.8	18
			N3/1B	8.6	26	TS 3B-bottom/bare soil	265.3	18
			N3/1C	68.4	13	TS 3C-top/biocrust	66.4	23
			N3/2A	0.5	28	TS 3C-top/bare soil	113.5	21
			N3/2B	14.3	32	TS 3C-bottom/biocrust	497.1	19
			N3/2C	111.1	17	TS 3C-bottom/bare soil	56.0	19
			N3/3A	1.1	21			
			N3/3B	0.5	26			
			N3/3C	7.3	15			
			N3/4A	BLD				
			N3/4B	142.4	15			
			N3/4C	0.6	29			

a *Means of the 3 technical replicates*.

b *Samples from Nubra Valley were all biocrust samples*.

c *BLD, below the limit of detection*.

## References

[B1] Angel R. (2012). Total nucleic acid extraction from soil. Protoc. Exch. 10.1038/protex.2012.046

[B2] Angel R. Claus P. Conrad R. (2012). Methanogenic archaea are globally ubiquitous in aerated soils and become active under wet anoxic conditions. ISME J. 6, 847–862 10.1038/ismej.2011.141 22071343 PMC3309352

[B3] Angel R. Conrad R. (2013). Elucidating the microbial resuscitation cascade in biological soil crusts following a simulated rain event. Environ. Microbiol. 15, 2799–2815 10.1111/1462-2920.12140 23648088

[B4] Angel R. Matthies D. Conrad R. (2011). Activation of methanogenesis in arid biological soil crusts despite the presence of oxygen. PLoS ONE 6:e20453 10.1371/journal.pone.0020453 21655270 PMC3105065

[B5] Bahl J. Lau M. C. Y. Smith G. J. D. Vijaykrishna D. Cary S. C. Lacap D. C. (2011). Ancient origins determine global biogeography of hot and cold desert cyanobacteria. Nat. Commun. 2, 163 10.1038/ncomms1167 21266963 PMC3105302

[B6] Banning N. Brock F. Fry J. C. Parkes R. J. Hornibrook E. R. C. Weightman A. J. (2005). Investigation of the methanogen population structure and activity in a brackish lake sediment. Environ. Microbiol. 7, 947–960 10.1111/j.1462-2920.2004.00766.x 15946291

[B7] Bates S. T. Berg-Lyons D. Caporaso J. G. Walters W. A. Knight R. Fierer N. (2011). Examining the global distribution of dominant archaeal populations in soil. ISME J. 5, 908–917 10.1038/ismej.2010.171 21085198 PMC3105767

[B8] Belnap J. (2003). Comparative structure of physical and biological soil crusts, in Biological Soil Crusts: Structure, Function and Management Ecological Studies, eds Belnap J. Lange O. L. (Berlin; Heidelberg: Springer), 177–191 10.1007/978-3-642-56475-8

[B9] Belnap J. Büdel B. Lange O. L. (2003). Biological soil crusts: characteristics and distribution, in Biological Soil Crusts: Structure, Function and Management Ecological Studies, eds Belnap J. Lange O. L. (Berlin; Heidelberg: Springer), 3–30 10.1007/978-3-642-56475-8

[B10] Belnap J. Eldridge D. (2003). Disturbance and recovery of biological soil crusts, in Biological Soil Crusts: Structure, Function, and Management Ecological Studies. eds Belnap J. Lange O. L. (Berlin; Heidelberg: Springer), 363–383

[B11] Brand W. A. (1996). High precision isotope ratio monitoring techniques in mass spectrometry. J. Mass Spectrom. 31, 225–235 10.1002/(SICI)1096-9888(199603)31:3<225::AID-JMS319>3.0.CO;2-L 8799274

[B12] Brankatschk R. Toewe S. Kleineidam K. Schloter M. Zeyer J. (2011). Abundances and potential activities of nitrogen cycling microbial communities along a chronosequence of a glacier forefield. ISME J. 5, 1025–1037 10.1038/ismej.2010.184 21124490 PMC3131848

[B13] Brune A. (2011). Methanogens in the digestive tract of termites, in (Endo)symbiotic Methanogenic Archaea Microbiology Monographs, ed Hackstein J. H. P. (Berlin; Heidelberg: Springer), 81–100

[B14] Bryant J. A. Lamanna C. Morlon H. Kerkhoff A. J. Enquist B. J. Green J. L. (2008). Microbes on mountainsides: contrasting elevational patterns of bacterial and plant diversity. Proc. Natl. Acad. Sci. U.S.A. 105, 11505–11511 10.1073/pnas.0801920105 18695215 PMC2556412

[B15] Büdel B. (2003). Synopsis: comparative biogeography of soil-crust biota, in Biological Soil Crusts: Structure, Function, and Management Ecological Studies, eds Belnap J. Lange O. L. (Berlin; Heidelberg: Springer), 141–152

[B17] Conrad R. (2005). Quantification of methanogenic pathways using stable carbon isotopic signatures: a review and a proposal. Org. Geochem. 36, 739–752 10.1016/j.orggeochem.2004.09.006

[B18] Conrad R. Claus P. Casper P. (2009). Characterization of stable isotope fractionation during methane production in the sediment of a eutrophic lake, Lake Dagow, Germany. Limnol. Oceanogr. 54, 457–471 10.4319/lo.2009.54.2.0457

[B19] Conrad R. Claus P. Casper P. (2010). Stable isotope fractionation during the methanogenic degradation of organic matter in the sediment of an acidic bog lake, Lake Grosse Fuchskuhle. Limnol. Oceanogr. 55, 1932–1942 10.4319/lo.2010.55.5.1932

[B20] Conrad R. Frenzel P. (2002). Flooded soils, in Encyclopedia of Environmental Microbiology, ed Bitton G. (New York, NY: John Wiley and Sons, Inc.), 1316–1333

[B21] Deppenmeier U. Mueller V. Gottschalk G. (1996). Pathways of energy conservation in methanogenic archaea. Arch. Microbiol. 165, 149–163 10.1007/BF01692856

[B22] Dridi B. Fardeau M.-L. Ollivier B. Raoult D. Drancourt M. (2012). *Methanomassiliicoccus luminyensis* gen. nov., sp. nov., a methanogenic archaeon isolated from human faeces. Int. J. Syst. Evol. Microbiol. 62, 1902–1907 10.1099/ijs.0.033712-0 22859731

[B23] Dvorský M. Doleܞal J. de Bello F. Klimešová J. Klimeš L. (2011). Vegetation types of East Ladakh: species and growth form composition along main environmental gradients. Appl. Veg. Sci. 14, 132–147 10.1111/j.1654-109X.2010.01103.x

[B24] Dvorský M. Doležal J. Kopeckı M. Chlumská Z. Janatková K. Altman J. (2013). Testing the stress-gradient hypothesis at the roof of the world: effects of the cushion plant thylacospermum caespitosum on species assemblages. PLoS ONE 8:e53514 10.1371/journal.pone.0053514 23326446 PMC3542354

[B25] Erkel C. Kube M. Reinhardt R. Liesack W. (2006). Genome of rice cluster I archaea - the key methane producers in the rice rhizosphere. Science 313, 370–372 10.1126/science.1127062 16857943

[B26] Evans R. D. Ehleringer J. R. (1993). A break in the nitrogen cycle in aridlands. Evidence from δ 15N of soils. Oecologia 94, 314–317 10.1007/BF00317104 28313666

[B27] Ferry J. G. (1994). Methanogenesis: Ecology, Physiology, Biochemistry and Genetics. New York, NY: Chapman and Hall

[B28] Fey A. Claus P. Conrad R. (2004). Temporal change of ^13^C-isotope signatures and methanogenic pathways in rice field soil incubated anoxically at different temperatures. Geochim. Cosmochim. Acta 68, 293–306 10.1016/S0016-7037(03)00426-5

[B29] Friedrich M. W. (2005). Methyl−coenzyme M reductase genes: unique functional markers for methanogenic and anaerobic methane−oxidizing archaea. Methods Enzymol. 397, 428–442 10.1016/S0076-6879(05)97026-2 16260307

[B30] Gangwar P. Alam S. I. Bansod S. Singh L. (2009). Bacterial diversity of soil samples from the western Himalayas, India. Can. J. Microbiol. 55, 564–577 10.1139/W09-011 19483785

[B31] Glissmann K. Conrad R. (2002). Saccharolytic activity and its role as a limiting step in methane formation during the anaerobic degradation of rice straw in rice paddy soil. Biol. Fertil. Soils 35, 62–67 10.1007/s00374-002-0442-z

[B32] Goevert D. Conrad R. (2009). Effect of substrate concentration on carbon isotope fractionation during acetoclastic methanogenesis by *Methanosarcina barkeri* and *M*. acetivorans and in rice field soil. .Appl. Environ. Microbiol. 75, 2605–2612 10.1128/AEM.02680-08 19251888 PMC2681706

[B33] Großkopf R. Janssen P. H. Liesack W. (1998). Diversity and structure of the methanogenic community in anoxic rice paddy soil microcosms as examined by cultivation and direct 16S rRNA gene sequence retrieval. Appl. Environ. Microbiol. 64, 960–969 9501436 10.1128/aem.64.3.960-969.1998 PMC106352

[B34] Hayes J. M. (1993). Factors controlling ^13^C contents of sedimentary organic compounds: principles and evidence. Mar. Geol. 113, 111–125 10.1016/0025-3227(93)90153-M

[B35] Hoehler T. M. Bebout B. M. Marais D. J. D. (2001). The role of microbial mats in the production of reduced gases on the early Earth. Nature 412, 324–327 10.1038/35085554 11460161

[B36] Janatková K. Řeháková K. Doležal J. Šimek M. Chlumská Z. Dvorskı M. (2013). Community structure of soil phototrophs along environmental gradients in arid Himalaya. Environ. Microbiol. 15, 2505–2516 10.1111/1462-2920.12132 23647963

[B36a] Lindström E. S. Langenheder S. (2012). Local and regional factors influencing bacterial community assembly. Environ. Microbiol. Rep. 4, 1–9 10.1111/j.1758-2229.2011.00257.x 23757223

[B37] Liu Y. Whitman W. B. (2008). Metabolic, phylogenetic, and ecological diversity of the methanogenic archaea. Ann. N.Y. Acad. Sci. 1125, 171–189 10.1196/annals.1419.019 18378594

[B38] Ludwig W. Strunk O. Westram R. Richter L. Meier H. Yadhukumar (2004). ARB: a software environment for sequence data. Nucleic Acids Res. 32, 1363–1371 10.1093/nar/gkh293 14985472 PMC390282

[B39] Lueders T. Chin K. Conrad R. Friedrich M. (2001). Molecular analyses of methyl−coenzyme M reductase α−subunit (mcrA) genes in rice field soil and enrichment cultures reveal the methanogenic phenotype of a novel archaeal lineage. Environ. Microbiol. 3, 194–204 10.1046/j.1462-2920.2001.00179.x 11321536

[B40] Middleton N. Thomas D. (1997). World Atlas of Desertification. London: UNEP

[B41] Nemergut D. R. Anderson S. P. Cleveland C. C. Martin A. P. Miller A. E. Seimon A. (2007). Microbial community succession in an unvegetated, recently deglaciated soil. Microb. Ecol. 53, 110–122 10.1007/s00248-006-9144-7 17186150

[B42] Nicholson M. Evans P. Joblin K. (2007). Analysis of methanogen diversity in the rumen using temporal temperature gradient gel electrophoresis: identification of uncultured methanogens. Microb. Ecol. 54, 141–150 10.1007/s00248-006-9182-1 17431710

[B43] Nicol G. W. Glover L. A. Prosser J. I. (2003). Molecular analysis of methanogenic archaeal communities in managed and natural upland pasture soils. Global Change Biol. 9, 1451–1457 10.1046/j.1365-2486.2003.00673.x

[B44] Nuebel U. Garcia-Pichel F. Muyzer G. (1997). PCR primers to amplify 16S rRNA genes from cyanobacteria. Appl. Environ. Microbiol. 63, 3327–3332 9251225 10.1128/aem.63.8.3327-3332.1997 PMC168636

[B45] Nüsslein B. Eckert W. Conrad R. (2003). Stable isotope biogeochemistry of methane formation in profundal sediments of Lake Kinneret (Israel). Limnol. Oceanogr. 48, 1439–1446 10.4319/lo.2003.48.4.1439

[B46] Offre P. Spang A. Schleper C. (2013). Archaea in biogeochemical cycles. Annu. Rev. Microbiol. 67, 437–457 10.1146/annurev-micro-092412-155614 23808334

[B47] Paul K. Nonoh J. O. Mikulski L. Brune A. (2012). “Methanoplasmatales,” Thermoplasmatales-related archaea in termite guts and other environments, are the seventh order of methanogens. Appl. Environ. Microbiol. 78, 8245–8253 10.1128/AEM.02193-12 23001661 PMC3497382

[B48] Peters V. Conrad R. (1995). Methanogenic and other strictly anaerobic bacteria in desert soil and other oxic soils. Appl. Environ. Microbiol. 61, 1673–1676 16535011 10.1128/aem.61.4.1673-1676.1995 PMC1388429

[B49] Poplawski A. B. Mårtensson L. Wartiainen I. Rasmussen U. (2007). Archaeal Diversity and community structure in a Swedish barley field: specificity of the Ek510r/(EURY498) 16S rDNA Primer. J. Microbiol. Methods 69, 161–173 10.1016/j.mimet.2006.12.018 17289189

[B50] Quast C. Pruesse E. Yilmaz P. Gerken J. Schweer T. Yarza P. (2013). The SILVA ribosomal RNA gene database project: improved data processing and web-based tools. Nucleic Acids Res. 41, D590–D596 10.1093/nar/gks1219 23193283 PMC3531112

[B51] Řeháková K. Chlumská Z. Doležal J. (2011). Soil cyanobacterial and microalgal diversity in dry mountains of Ladakh, NW Himalaya, as related to site, altitude, and vegetation. Microb. Ecol. 62, 337–346 10.1007/s00248-011-9878-8 21643700

[B52] Roy R. Klueber H. D. Conrad R. (1997). Early initiation of methane production in anoxic rice soil despite the presence of oxidants. FEMS Microbiol. Ecol. 24, 311–320 10.1111/j.1574-6941.1997.tb00448.x

[B53] Sakai S. Imachi H. Hanada S. Ohashi A. Harada H. Kamagata Y. (2008). *Methanocella paludicola* gen. nov., sp. nov., a methane-producing archaeon, the first isolate of the lineage “Rice Cluster I,” and proposal of the new archaeal order Methanocellales ord. nov. Int. J. Syst. Evol. Microbiol. 58, 929–936 10.1099/ijs.0.65571-0 18398197

[B54] Scavino A. F. Ji Y. Pump J. Klose M. Claus P. Conrad R. (2013). Structure and function of the methanogenic microbial communities in Uruguayan soils shifted between pasture and irrigated rice fields. Environ. Microbiol. 15, 2588–2602 10.1111/1462-2920.12161 23763330

[B55] Shindell D. T. Faluvegi G. Koch D. M. Schmidt G. A. Unger N. Bauer S. E. (2009). Improved attribution of climate forcing to emissions. Science 326, 716–718 10.1126/science.1174760 19900930

[B57] Sjoeling S. Cowan D. A. (2003). High 16S rDNA bacterial diversity in glacial meltwater lake sediment, Bratina Island, Antarctica. Extremophiles 7, 275–282 10.1007/s00792-003-0321-z 12910387

[B58] Soule T. Anderson I. J. Johnson S. L. Bates S. T. Garcia-Pichel F. (2009). Archaeal populations in biological soil crusts from arid lands in North America. Soil Biol. Biochem. 41, 2069–2074 10.1016/j.soilbio.2009.07.023

[B59] Stamatakis A. (2006). RAxML-vi-hpc: maximum likelihood-based phylogenetic analyses with thousands of taxa and mixed models. Bioinformatics 22, 2688–2690 10.1093/bioinformatics/btl446 16928733

[B60] Steinberg L. M. Regan J. M. (2008). Phylogenetic comparison of the methanogenic communities from an acidic, oligotrophic fen and an anaerobic digester treating municipal wastewater sludge. Appl. Environ. Microbiol. 74, 6663–6671 10.1128/AEM.00553-08 18776026 PMC2576706

[B61] Steven B. Gallegos-Graves L. V. Yeager C. M. Belnap J. Evans R. D. Kuske C. R. (2012). Dryland biological soil crust cyanobacteria show unexpected decreases in abundance under long-term elevated CO_2_. Environ. Microbiol. 14, 3247–3258 10.1111/1462-2920.12011 23116182

[B62] West A. E. Schmidt S. K. (2002). Endogenous methanogenesis stimulates oxidation of atmospheric methane in alpine tundra soil. Microb. Ecol. 43, 408–415 10.1007/s00248-001-1049-x 12043000

[B63] Zinder S. H. (1993). Physiological ecology of methanogens, in Methanogenesis: Ecology, Physiology, Biochemistry and Genetics, ed Ferry J. G. (London: Chapmann and Hall), 128–208

